# Prevalence of typhoid fever and its associated factors among febrile patients visiting Arerti Primary Hospital, Amhara Region, north east Ethiopia

**DOI:** 10.3389/fpubh.2024.1357131

**Published:** 2024-08-16

**Authors:** Genet Muche, Asmamaw Tesfaw, Fekade Demeke Bayou

**Affiliations:** ^1^Department of Biology, Debre Berhan University, Debre Berhan, Ethiopia; ^2^Department of Epidemiology and Biostatistics, School of Public Health, Colleges of Medicine and Health Science, Wollo University, Dessie, Ethiopia

**Keywords:** prevalence, typhoid fever, Arerti, Ethiopia, associated factors

## Abstract

**Background:**

Typhoid fever is one of the major public health concerns in developing countries, including Ethiopia. Understanding the burden and factors contributing to the transmission and development of the disease is crucial to applying appropriate preventive and therapeutic interventions.

**Objective:**

To assess the prevalence of typhoid fever and its associated factors among febrile patients visiting Arerti Primary Hospital from 1 March to 30 May 2022.

**Methods:**

A facility-based cross-sectional study was employed among 326 febrile patients visiting Arerti Primary Hospital for health services. The data were collected using laboratory procedures (widal test) and a structured interviewer-administered questionnaire. The data were entered using Epi Data version 3.1 and analyzed by SPSS version 25. Logistic regression was used to determine associations between variables. *P*-value < 0.05 and adjusted odds ratio with 95% confidence interval were used to measure the presence and strength of associations.

**Results:**

In this study, of the total 317 cases that participated, the majority (64.4%) of them were males with age ranges from 13 to 63 years. The overall prevalence of positive antigen tests for typhoid infection was 30.0% (95% CI: 25.0%−35.3%). About 66.9% of the study participants had good knowledge, 75.7% had favorable perception, and 42.3% had good infection prevention practice. Being unemployed [AOR = 7.57, 95% CI (1.98, 28.93)], being a farmer [AOR = 2.73, 95% CI (1.01, 7.41)], and having a body mass index (BMI) below 18.5 kg/m^2^ [AOR = 5.12, 95%CI (2.45, 10.68)] were significantly associated with typhoid fever infection.

**Conclusion:**

The prevalence of typhoid fever among febrile patients was high. Typhoid fever infection was significantly associated with occupational status (being unemployed and being a farmer) and lower BMI. The level of knowledge, perception, and practice of typhoid fever infection prevention were found inadequate. Therefore, behavioral change interventions are needed at the community level.

## Introduction

Typhoid fever is a life-threatening acute febrile illness caused by *Salmonella typhi and paratyphi* (*with a* 10:1 case ratio) (1–3). After ingestion in contaminated food or water, typhoid organisms pass through the pylorus, reach the small intestine, penetrate the mucosal epithelium, cause acute infection, and are released into the bloodstream by lymph drainage from mesenteric nodes. Incubation periods ranging from 3 days to more than 60 days have been reported (1). After infection with the bacteria, symptoms usually start 8–14 days later for typhoid fever and 1–10 days later for paratyphoid fever (3). The clinical presentation of typhoid fever varies from a mild illness with low-grade fever, malaise, and a slight dry cough to a severe clinical picture with abdominal discomfort and multiple complications. Depending on the clinical setting and the quality of available medical care, a significant portion of typhoid patients may develop serious complications like intestinal perforation, hemorrhage, hepatitis, altered mental status (confusion and delirium), etc. (1, 3). The definitive diagnosis of typhoid fever depends on the isolation of *S. typhi* from blood, bone marrow, or a specific anatomical lesion. Blood culture is the mainstay of the diagnosis of this disease (1). Transmission of typhoid fever can be prevented by maintaining food safety, safe water supply, proper sanitation, vaccination, and health education to create public awareness and induce behavioral change after identifying socio-demographic, cultural, and economic risk factors contributing to the transmission of the disease (1, 3).

Globally, there were more than 14 million cases of typhoid and paratyphoid fevers, with an estimated 136,000 deaths. Moreover, typhoid and paratyphoid fevers were responsible for 9.8 million disability-adjusted life years (DALYs) in 2017 (4, 5). In Africa, typhoid fever remains a higher endemic tropical disease of public health significance due to its faster transmission rates (6, 7). The reported figures on typhoid fever cases show that the trend of typhoid-related morbidity is in rapid increment over time in Africa. Outbreaks of typhoid fever were reported in 15 countries (6). The estimated case fatality rate among non-surgical patients was 5.4% for the Africa region, which is six times higher than that of the Asia region (5). The burden of the disease was more pronounced in sub-Saharan Africa (SSA), with 7.2 million cases of typhoid fever annually and an incidence rate of 762 per 100,000 person-years. Recent studies show that almost all regions of SSA are tending toward high incidence rates, especially Central and Western Africa (7).

Typhoid and paratyphoid fever was endemic in many countries in South Asia, sub-Saharan Africa, and East Asia and the Pacific (8–10). In Africa, a study done in Kaduna State and South-East Nigeria showed that the prevalence of typhoid fever was 33.3% and 14.1%, respectively (11, 12). In the Democratic Republic of Congo and South Africa, the annual incidence of typhoid fever ranged from 1.7 to 9.1% and 0.11 to 0.39 per 100,000 populations, respectively (13, 14). In Cameroon, the prevalence of typhoid fever was 68.75% (15), while it was found at 943 per 100,000 in Ghana (16). In Ethiopia, limited studies found that 13.2, 10, and 25.7% of the study population in the Afar Region, West Wollega Zone, and Injibara, Northwest Ethiopia, respectively, had *S. Typhi* infection (17–19). In general, the estimated pooled prevalence of typhoid fever from blood and stool culture diagnosis was 3% and Widal test examination 33% (20). Socio-demographic and clinical conditions including the duration and severity of typhoid fever disease, sex, age, personal and environmental hygiene (11, 21), income (15, 16), sharing food, ownership/utilization of toilets (22, 23), residence (24), educational level, and misconception about the transmission of typhoid fever (18, 25–27) were found to be significantly associated with typhoid prevalence.

To combat the impact of typhoid fever on the health, economic, and social activities of the community, there should be clear information regarding the magnitude of the disease and its socio-demographic, economic, cultural, and geographic risk factors contributing to the transmission and development of the disease. To evaluate the impact of this infectious disease in a particular area, one must be aware of the prevalence of typhoid fever. For public health officials to deploy focused interventions and allocate resources efficiently, this information is essential. Understanding associated risk factors facilitates understanding the mechanisms underlying the transmission of typhoid fever. This information can direct the creation of approaches to stop the spread, like strengthening immunization campaigns and enhancing food safety, water quality, sanitation, and hygiene. Understanding the prevalence and risk factors helps medical professionals diagnose and treat patients more accurately. It can result in faster, more precise patient care, better recovery rates, and a decrease in typhoid fever-related complications or deaths. However, such relevant information is limited in Ethiopia, particularly in the study area. Hence, this study was aimed at assessing the prevalence of typhoid fever and its associated factors among febrile patients attending Arerti Primary Hospital.

## Methods and materials

### Study setting, design, and period

This study was conducted at Arert Primary Hospital located in Minjar Shenkora woreda, Amhara Regional State, Ethiopia. Minjar Shenkora Woreda is located 130 km from Addis Ababa. The woreda is populated with an estimated number of 156,040 people (80,729 male) and (75,311 female) (28). There are five health centers, 27 health posts, and one primary hospital providing health services for the catchment population. A facility-based cross-sectional study was conducted from March 01 to May 30, 2022.

### Population

#### Source population

The source population was all febrile patients attending Arerti Primary Hospital during the study period.

#### Study population

Selected febrile patients clinically suspected for typhoid fever in the study period were considered as the study population.

### Inclusion and exclusion criteria

#### Inclusion criteria

All febrile patients clinically suspected for typhoid fever in the study period were included in the study.

#### Exclusion criteria

Patients with impaired mental status (unconscious) during the study period were excluded.

### Sample size determination

The sample size for this study was determined using a single population proportion formula by taking the proportion (*p*) of typhoid fever of 25.7% from the previous study (18), the confidence level of 95%, the marginal error of 5%, and adding 10% non-response rate. It was calculated as follows:


$$\begin{array}{l} n=\frac{Z\alpha_{/\;2}2\;\left(P\right)\left(1-P\right)}{{\left(d\right)}^{2}}\;n=\frac{{\left(1.96\right)}^{2}\left(0.26\right)\left(0.74\right)}{{\left(0.05\right)}^{2}}. \end{array}$$

After adding 10% non-response rate, the total sample size for this study was 326 individuals.

### Sampling technique

A systematic random sampling technique was used to select study participants. First, we classified the hospitals into four outpatient departments (OPD) as OPD1, OPD2, OPD3, and OPD4. Then patients were distributed into each OPD. Then, systematic random sampling was used to select respondents from each OPD. Sampling interval (*K*) was calculated by dividing total estimated febrile patients visiting the hospital during the study period (*N*) by the sample size (*n*). It gave a value of *K* = 11, hence the first individual was selected by lottery method, and then the next participants were chosen in every 11^th^ interval until the total sample size was reached.

### Data collection, clinical sample processing, and serological testing

All febrile patients visiting Arerti Primary Hospital outpatient department (OPD) during the study period were examined clinically by physicians or health professionals. Patients presenting with fever (axillary temperature >37.5°C) and any of the complaints like abdominal pain or discomfort, headache, constipation, or diarrhea were requested for a Widal test. After getting informed consent, data on individual and household socio-demographic data such as age, sex, family size, occupation, education, residence, marital status, dietary habits, safe water source, and toilet access were collected by face-to-face interview using a structured questionnaire. After the interview, a 5 ml blood specimen was collected from each study participant in the laboratory department using a test tube without anticoagulant. The blood samples were collected by puncturing superficial veins (mainly from median cubital veins) of the upper limb using a 5 cc needle. The collected blood samples were processed to get serum, and the Widal slide agglutination tests were done to detect *S. Typhi* using known *S. Typhi* flagella (H) and somatic (O) antigens.

The slide-agglutination tests were performed as per the manufacturer's instruction. Briefly, the antigen vials were gently mixed with saline by an aspirate dropper multiple times to make a thorough mixture. A 50 μl serum sample was added to a row of circles on the test card. Drops (one drop each) of positive and negative control sera were dispensed into respective circles. A drop of the appropriate well-shaken suspension of the antigen was added to each circle next to each sample to be tested and mixed with the content of each circle with a disposable stirrer and spread over the entire area enclosed by the ring with separate applicators for each mixture. After that, the slides were shaken gently by hand or using a mechanical rotator (100 rpm) for 1 min. Finally, the test was observed immediately under a suitable light source for any degree of agglutination, and qualitative results were recorded.

### Study variables

#### Dependent variable

Typhoid fever status (positive or negative).

#### Independent variable

Independent variables include socio-demographic variables (like sex, age, educational status, occupational status, marital status, residence, sources of water, etc.), history or current chronic medical illness and recurrent infection, knowledge about TF cause, route of transmission, and preventive measures to be taken, perception about TF cause, route of transmission, and preventive measures to be taken, and practice of febrile patients to prevent TF infection.

### Operational definitions

#### Case definition of typhoid fever

According to the Ethiopian Ministry of Health, typhoid fever is defined as: A probable or suspected case of typhoid fever: A patient with documented fever (38°C and above) for at least 5 days prior to presentation with a rising trend AND having no other focus to explain the cause of the fever (e.g., malaria, meningitis, pneumonia, abscess, pyelonephritis, etc.). A confirmed case of typhoid fever: A patient with persistent fever (38°C or above) lasting three or more days and S. Typhi isolated on culture (blood, bone marrow culture, stool, or urine) (29).

#### Knowledge about typhoid fever

Patients' knowledge about typhoid fever was assessed using 11 yes or no item questions concerned with the cause, mode of transmission, and preventive measures of typhoid fever. Each correct answer was scored “1” and the incorrect answer was “0,” with the sum ranging from 0 to 11. The mean score was calculated to determine their level of knowledge, and participants who scored the mean and above were considered to have good knowledge, while those scored below the mean score were regarded as having poor knowledge.

#### Perception toward typhoid fever

Patients perception of being exposed to or at risk of acquiring typhoid fever, susceptibility, preventability, and severity of typhoid fever were asked to assess their perception toward typhoid fever.

#### Practice of typhoid infection prevention

A total of 14 practice-related questions were used to assess participants' practice of typhoid infection prevention. The average score was calculated to determine their level of prevention practice, and participants who scored the mean and above were considered to have good prevention practice, while those scored below the mean score were regarded as having poor prevention practice.

### Data quality control

The questionnaire was pretested on 5% of the sample population in Arerti health center. During the pre-test, the questionnaire was evaluated for its clarity, sensitivity, and cultural acceptability. Confusing questions were identified based on the obtained results from the pretest, and necessary modifications have been made to the questions. Training was given for data collectors on the portion of the questionnaire and data collection procedures. The questionnaire was translated from English to Amharic by language experts to ease the data collection process. During data collection, on-site supervision and technical assistance were provided by the principal investigator. Finally, the questionnaire was checked daily for its completeness and consistency by the principal investigator, and any incomplete information was excluded from analysis after data collection.

### Data processing and analysis

The collected data were entered into the computer using Epi Data version 3.1 and then exported to SPSS version 25 for further analysis. The data was cleaned, edited, sorted, and coded before analysis. Descriptive statistics such as mean, median, and standard deviation were carried out for continuous variables. Participants' responses to knowledge and practice-related questions were coded, scored, summed, and averaged. Eleven (11) questions were asked for the participants to assess their level of knowledge about typhoid fever. Those who answered the correct answer were scored “1” and “0” if not. The summed scores ranged from 0 to 11. The mean (average) value of scores was calculated (which was 9.15) and used to categorize participants's level of knowledge. Hence, respondents who scored below the 9.15 in the knowledge-related question were regarded as having poor knowledge, while those who scored 9.15 and above were considered to have good knowledge. The same method was applied to assess the level of typhoid fever infection prevention practice. Bivariable and multivariable logistic regression models were fitted to determine factors associated with the occurrence of typhoid fever. A *P*-value of < 0.05 was used to test statistical significance and adjusted odds ratio (AOR) with its 95% CI as a measure of strength of association between variables.

## Result

### Socio-demographic characteristics of participants

In this study, about 317 febrile patients participated, giving a response rate of 97.2%. Out of which the majority (64.4%) of them were males. The age of study participants ranged from 13 to 63 years, with a mean ± SD of 34.59 to 12.07 years. Nearly one-fourth of the participants were university degree holders. Regarding their occupational status, more than one third of them (35%) were farmers, followed by merchants 65 (20.5%) (Table 1).

**Table 1:** Socio-demographic characteristics of febrile patients visiting APH, Arerti town, Ethiopia, 2022.

**Characteristics**	**Categories**	**Frequency (*n*)**	**Percentage (%)**
Sex of the respondents	Male	204	64.4
	Female	113	35.6
Age in years of the respondents	<20	40	12.6
	20–29	86	26.5
	30–39	100	31.5
	40–49	53	16.7
	50–59	22	6.9
	≥60	18	5.7
Educational status of the respondents	Cannot read and write	34	10.7
	Can read only	13	4.1
	Can read and write	67	21.1
	Completed primary education	34	10.7
	Secondary education	70	22.1
	Diploma	24	7.6
	Degree and above	75	23.7
Average monthly income in birr	<5,000	142	44.7
	5,000–10,000	166	52.4
	10,001–15,000	4	1.3
	15,001–20,000	5	1.6
Religion of the respondents	Orthodox	260	82
	Protestant	10	3.2
	Muslim	47	14.8
Marital status of the respondents	Single	120	37.8
	Married	193	60.9
	Divorce/widowed	4	1.3
Occupation of respondents	Farmer	111	35
	Merchant	52	16.4
	Government employ	65	20.5
	Student	74	23.3
	Other	15	4.8

Concerning the type of water sources, most (89.6%) of the study participants were getting pipe water. For more than half (53.3%) of the respondents, the time taken to reach the water sources was <5 min. About 26 (8.2%) of the study participants had any chronic health problem (comorbidities), and hypertension was the most frequently seen comorbidity (Table 2).

**Table 2:** Source of water and comorbidity status of febrile patients visiting Arerti Primary Hospital, Ethiopia, 2022.

**Variables**	**Categories**	**Frequency**	**Percent**
Source of water	Pipe line	284	89.6
	Spring	33	10.4
Traveling time to get water source in minutes	<5	169	53.3
	5–10	63	19.9
	11–15	39	12.3
	16–20	8	2.5
	21–25	9	2.8
	≥26	15	4.7
Weight of respondents in kg	<50	7	2.2
	50–65	169	53.3
	66–75	92	29.1
	76–85	40	12.6
	≥86	9	2.8
Presence of chronic diseases	Yes	26	8.2
	No	291	91.8
What type of chronic disease do you have?	Hypertension	13	50.0
	Diabetes mellitus	9	34.6
	Others	4	1.3

### Knowledge, perception, and preventive practice of typhoid infection

#### Knowledge about typhoid fever

The patients' knowledge about preventive measures, transmission route, and signs and symptoms of TF was assessed using 11 yes/no questions. The scores were ranging from zero (0) to eleven (11) with a mean of 9.15. Participants who scored more than or equal to the mean were considered to have good knowledge. The overall proportion of participants who had good knowledge about typhoid fever was 66.9% (212). Most (91.2%) of the respondents know that fever can be a sign of typhoid fever infection (Table 3).

**Table 3:** Febrile patient knowledge about the preventive measure of TF, route of transmission, and clinical presentation of TF in APH, Arerti town, Ethiopia, 2022.

**Knowledge assessment items**	**Response**	**Frequency**	**Percentage**
TF is caused by microorganisms	No	45	14.2
	Yes	272	85.8
Fever is one sign of TF	No	28	8.8
	Yes	289	91.2
Typhoid fever can kill infected individuals	No	27	8.5
	Yes	290	91.5
Typhoid fever is transmitted from an infected person to other healthy persons	No	65	20.5
	Yes	252	79.5
Typhoid fever is transmitted by eating contaminated food	No	46	14.5
	Yes	271	85.5
Typhoid fever is transmitted by drinking contaminated water	No	58	18.3
	Yes	259	81.7
Typhoid fever carriers can act as a source of TF infection	No	110	34.7
	Yes	207	65.3
Hand washing before meals can prevent TF infection	No	47	14.8
	Yes	270	85.2
Hand washing after the toilet can prevent TF infection	No	56	17.7
	Yes	261	82.3
Typhoid fever can be prevented by proper food cooking	No	53	16.7
	Yes	264	83.3
Typhoid fever can be prevented by washing table fruits and vegetables	No	52	16.4
	Yes	265	83.6
Knowledge of study participants about typhoid fever infection	Poor knowledge	105	33.1
	Good knowledge	212	66.9

#### Participants perception toward typhoid fever

In this study, the majority (64.4%) of the study participants perceived themselves as they were exposed (at risk) to typhoid fever infection. The mean score of the perception-related question was 3.0; participants who scored the mean and above were regarded as having positive perception. Three-fourths (75.7%) of respondents had scored the mean and above out of four perception-related questions, which implies that they had a good perception about the TF disease source of infection, route of transmission, and its preventive measures (Table 4).

**Table 4:** Perception of the study participants about the preventive measure of TF and route of transmission of TF in APH, Arerti town, Ethiopia, 2022.

**Perception assessment items**	**Response**	**Frequency**	**Percent**
Do you believe that you are exposed to TF infectious sources?	No	113	35.6
	Yes	204	64.4
Do you think that TF is a preventable disease?	No	72	22.7
	Yes	245	77.3
Do you think that washing your hands before a meal can prevent you from getting a TF infection?	No	47	14.8
	Yes	270	85.2
Do you think consuming raw fruit and vegetables can expose you to TF infection?	No	81	25.6
	Yes	236	74.4
Perception of the study participants about the preventive measure of TF	Negative perception	77	24.3
	Positive perception	240	75.7

#### Participants' practice to typhoid fever infection prevention

In this study, participants' practice of typhoid fever infection prevention was assessed using 14 diverse closed-ended questions. The minimum and maximum scores were 3.0 and 13.0, with a mean of 7.42. Of those 317 febrile patients, 134 (42.3%) were scored the mean and above on the TF infection practice-related questions. More than half (57.7%) of the study participants did not have proper waste disposal practice, and only 11.0% were adequately practicing hand washing practice after using the toilet (Table 5).

**Table 5:** Febrile patients' practices to prevent TF infection among febrile patients who were visiting APH, Arerti town, Ethiopia, 2022.

**Practice assessment items**	**Response**	**Frequency**	**Percentage**
Do you consume raw meat in the last month?	Yes	138	43.5
	No	179	56.5
Do you consume raw milk in the last month?	Yes	119	37.5
	No	198	62.5
Do you use pipeline water?	Yes	312	98.4
	No	5	1.6
Do you have a garbage can for waste collection at your home?	Yes	134	42.3
	No	183	57.7
Do you have a toilet and use it properly?	Yes	289	91.2
	No	28	8.8
Do you usually wash your hands using soap after toilet usage?	Always	35	11.0
	Sometimes	282	89.0
Do you have an animal living in the same house?	Yes	86	27.1
	No	231	72.9
Do you store food for later consumption after use?	Yes	268	84.5
	No	49	15.5
Do you share food from the same plate?	Yes	258	81.4
	No	59	18.6
Do you eat uncooked food?	Never	15	4.7
	Some times	302	95.3
Do you wash your hands before preparing food?	Always	54	17.0
	Some times	263	83.0
Are you smoking a cigarette?	Yes	9	2.8
	No	308	97.2
Are you drinking alcohol?	Yes	114	36.0
	No	203	64.0
Did you get a health education?	Yes	192	60.6
	No	125	39.4
Overall practice for typhoid infection prevention	Poor practice	183	57.7
	Good practice	134	42.3

### Prevalence of typhoid fever among febrile patients

**Figure 1:**
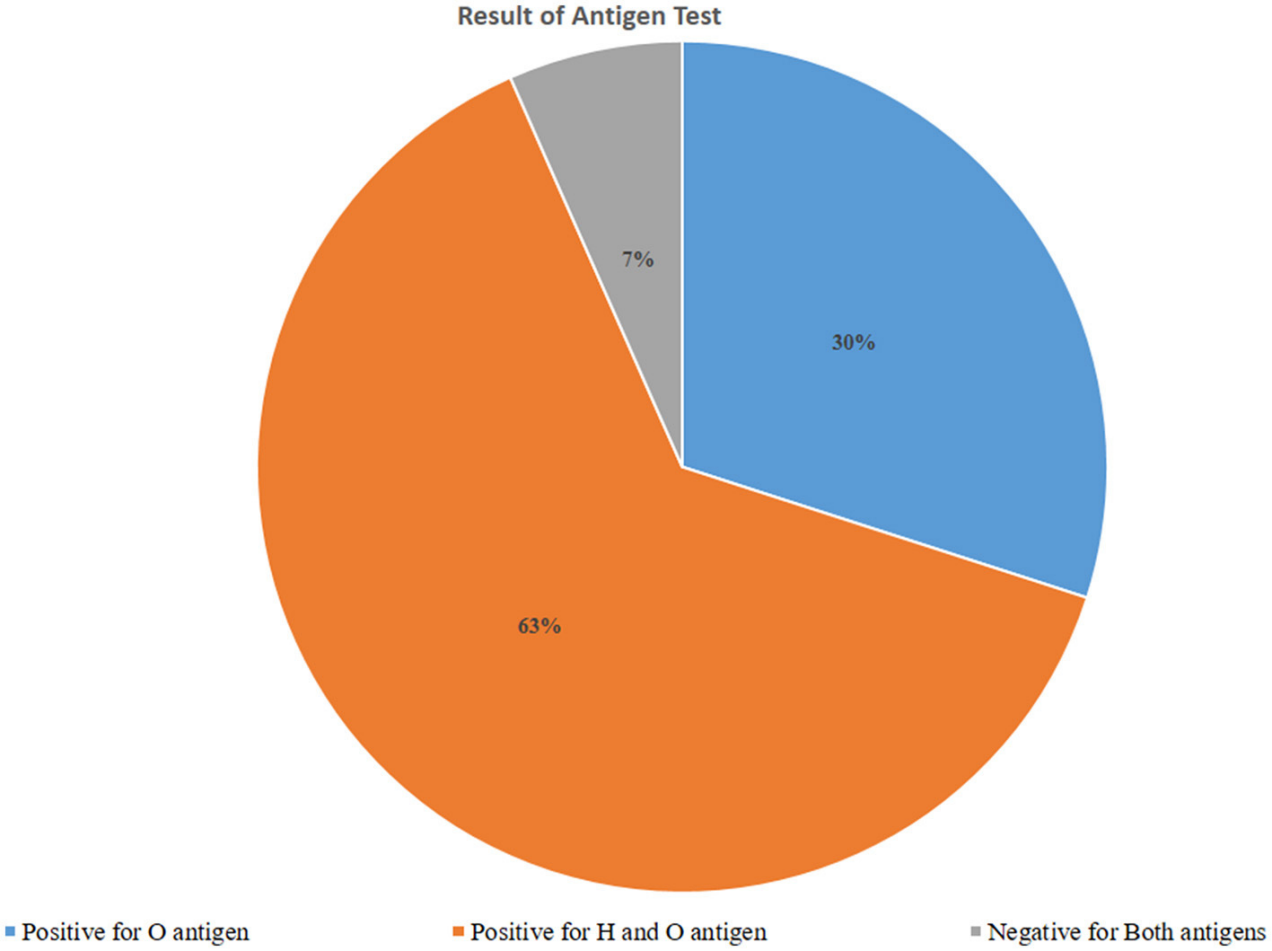
Typhoid fever infection based on an antigen test using the Widal test among febrile patients visiting Arerti Primary Hospital, Amhara, 2022.

Of those 317 febrile patients who got the Widal diagnostic test, O antigen was detected among 95 (30.0%) of the respondents. Both H antigen and O antigen were detected among 201 (63.4%) of the respondents. Since the flagellated H antigen may not indicate the current typhoid fever infection, we didn't rely on this antigen result to estimate typhoid fever infection status. Hence the overall prevalence of typhoid fever in the study setting was 30.0%, 95% CI (25.0%−35.3%; Figure 1).

### Factors associated with typhoid fever infection

A bi-variable and multivariable logistic regression model was fitted to assess the relationship between dependent and independent variables. Accordingly, preliminary assessment was done using bivariable analysis to select the candidate variables for multivariable logistic regression, the final model. Variables including sex of the respondents, occupational status, educational status, marital status, body mass index, knowledge about typhoid infection, perception, and practice of typhoid infection prevention were moved to the multivariable logistic regression model. After controlling the effect of confounding variables, occupational status being unemployed [AOR = 7.57, 95% CI (1.98, 28.93)], being a farmer [AOR = 2.73, 95% CI (1.01, 7.41)], and having a BMI <18.5 kg/m^2^ [AOR = 5.12, 95% CI (2.45, 10.68)] were statistically significantly associated with typhoid fever infection. In this study, unemployed study participants were more than seven times more likely to have typhoid fever infection than merchants. Likewise, as compared with the same group, farmers were nearly three times more likely to have a positive antigen test for typhoid infection. Moreover, study participants whose body mass index (BMI) was <18.5 kg/m^2^ were more than five times more likely to have typhoid infection as compared with their counterparts (Table 6).

**Table 6:** Multivariable analysis of factors significantly associated with the prevalence of TF among febrile patients visiting APH at Arerti town, Amhara, Ethiopia, 2022.

**Variables**		**TF test results**		**COR (95% CI)**	**AOR (95% CI)**	***P*-value**
		**Positive**	**Negative**			
Sex of the respondent	Male	63	141	1.13 (0.68, 1.88)	0.99 (0.55, 1.76)	0.963
	Female	32	81	1	1	
Occupation of the respondent	Unemployed	31	53	1.38 (0.67, 2.83)	7.57 (1.98, 28.93)	**0.003**
	Farmer	34	77	1.04 (0.52, 2.09)	2.73 (1.01, 7.41)	**0.048**
	Gov't employee	13	52	0.59 (0.26, 1.35)	0.82 (0.23, 2.91)	0.755
	Merchant	17	40	1	1	
Educational status	Uneducated	24	90	0.71 (0.38, 1.34)	0.35 (0.09, 1.38)	0.132
	Primary/secondary	44	60	1.96 (1.09, 3.52)	1.12 (0.44, 2.84)	0.807
	Diploma & above	27	72	1	1	
Marital status	Married	58	135	0.99 (0.61, 1.62)	0.39 (0.15, 1.04)	0.060
	Unmarried	37	87	1	1	
BMI	<18.5	18	104	3.77 (2.12, 6.71)	5.12 (2.45, 10.68)	**<0.01**
	18.5 and above	77	118	1	1	
Practice categorized	Poor practice	61	122	0.68 (0.41, 1.12)	1.06 (0.57, 1.98)	0.845
	Good practice	34	100	1	1	
Perception toward typhoid fever prevention	Negative perception	19	58	1.42 (0.80, 2.54)	1.52 (0.59, 3.94)	0.384
	Positive perception	76	164	1	1	
Knowledge about TF	Poor knowledge	24	81	1.70 (0.99, 2.91)	0.69 (0.24, 1.95)	0.482
	Good knowledge	71	141	1		

Bold values indicate statistically significant (*p* < 0.05).

## Discussion

In this study, the prevalence of typhoid fever and its associated variables were assessed among febrile patients visiting Arerti Primary Hospital for health services. Accordingly, the overall prevalence of positive antigen tests for typhoid infection was 30% (95% CI: 25.0%−35.3%). Nearly two-thirds (66.9%) of the study participants had good knowledge about the cause, transmission, and prevention methods of typhoid fever infection. Similarly, three-fourths (75.7%) of the participants had favorable perceptions for typhoid fever infection prevention. However, only 42.3% of them had good infection prevention practices. Being unemployed, a farmer, and having a BMI <18.5 kg/m^2^ were significantly associated with typhoid fever infection.

The figure reported in this study, 30.0%, was in line with the findings reported in Pakistan (29.34%) (11), China (29.3%) (8), and Kaduna State, Nigeria (33.3%) (12). The observed concordance might be due to similarity in study population and method of assessment. For instance, the study conducted in Pakistan assessed the prevalence of typhoid fever among febrile patients using widal tests, which applied a similar approach with our study. The study conducted in Injibara, Northeast Ethiopia, reported that the prevalence of typhoid fever was 25.7%, which was consistent with our study finding (18). This consistency might be justified by the similarity of the two studies in terms of study setting, socio-demographic factors, and methods used to measure the outcome variable.

However, the current figure was higher than findings reported in the Democratic Republic of Congo (9.1%) (14), Lagos, Nigeria (15.9%) (30), and Zaria (4.9% among children) (31). This discrepancy might be due to the difference in study population, time of the study, laboratory modalities used, health facility settings, and socio-demographic characteristics of the study participants. For example, the study conducted in Zaria included only children (<15 years old; nearly one third were under 5 years old) as study participants; this group would most probably be affected by other etiologic agents like the Rota virus rather than typhoid fever. Moreover, the study conducted in Lagos, Nigeria, used culture to detect the salmonella species, whereas the Widal test was used in this study. The difference in measuring tool (diagnostic tool) might contribute to the observed discrepancy of the outcome variable.

In Ethiopia, studies conducted in the Afar (17), West Wollega, and Oromia (19) regions reported that the prevalence of typhoid fever was 13.2 and 10.0%, respectively. Which is lower as compared to the figure found by this study (30.0%). The probable reason for the observed difference in terms of typhoid fever prevalence could be a difference in study settings; the above studies were community-based studies while ours was a facility based study. The community-based studies identified the prevalence of typhoid fever among a healthy population, whereas the facility-based studies assessed the same outcome among patients manifested with apparent clinical manifestations. Hence, this might end up with a significant difference in the disease magnitude.

On the other hand, the finding from this study was lower as compared with the figures found by the studies conducted in Littoral, Cameroon (68.75%) (16), Akure, Nigeria (80.5%) (32), Ghana (66.9%) (16), and Karu, Nigeria (69.6%) (33). This discordance might result from discrepancies in study populations, sample size, study settings, and socio-demographic variability of the study participants. For instance, the study conducted in Cameroon included a small sample size of study participants who were clinically diagnosed with typhoid fever. This might overestimate the outcome as included already clinically diagnosed cases for the confirmation purpose. Moreover, in Ethiopia, studies done in Arba Minch, SNNPR, Ethiopia (25) found higher prevalence of typhoid fever (47.37% among males and 52.3% among females) as compared with the reported figure by this study. This difference might be due to differences in study designs, study population, measurement tools, and environmental determinants of health. The mentioned study was a type of trend analysis using secondary data (health facility records), which assessed typhoid fever diagnosed by using both clinical and laboratory diagnosis methods. This measurement difference might be the reason for the observed discordance.

In this study, being unemployed, a farmer, and having a lower body mass index (BMI) were significantly associated with the occurrence of typhoid fever infection. Although not supported by previous studies, unemployed (daily labor) and farmers would be more likely to be involved in risky conditions for typhoid fever transmission. Since most farmers reside in rural areas, they might also face inadequate access to safe water, and late healthcare-seeking behavior may speed transmission. Having a lower body mass index (BMI) may indicate a state of undernutrition; hence, undernourished individuals might be highly susceptible for infection and other comorbidities. This study might have the following limitation: since we conducted it at a single health facility, the result may not be generalizable to the whole community.

## Conclusions and recommendations

The prevalence of TF among febrile patients in the study area was high. Typhoid fever infection was significantly associated with occupational status (being unemployed and being a farmer) and body mass index (BMI). The level of knowledge, perception, and practice for typhoid fever infection prevention was not adequate. Our findings may inform health planners and administrators in developing relevant interventions that promote TF preventive practice toward TF disease among the population.

## Data Availability

The original contributions presented in the study are included in the article/supplementary material, further inquiries can be directed to the corresponding author.
